# Dysregulated circRNA–miRNA–mRNA networks reveal stage-specific mRNA expression changes in Parkinson’s disease

**DOI:** 10.1186/s13041-025-01262-2

**Published:** 2025-12-01

**Authors:** Yulan Gao, Konii Takenaka, Kristina Santucci, Grace Lindner, Si-Mei Xu, Yuning Cheng, Michael Janitz

**Affiliations:** School of Biotechnology and Biomolecular Sciences, University of New South Wales, Sydney, NSW 2052 Australia

**Keywords:** Parkinson’s disease, circRNA-miRNA-mRNA, Uniquely expressed circRNAs, RNA-seq, circPRDM2, circHSH2D, CACNG8, PLXNB1

## Abstract

Parkinson’s disease (PD) is a progressive neurodegenerative disorder marked by the loss of dopaminergic neurons and widespread transcriptomic dysregulation across disease stages. Patients commonly exhibit motor symptoms such as tremors, rigidity, and bradykinesia, alongside non-motor symptoms including depression and cognitive decline. While previous research has largely focused on protein-coding genes, growing attention is being directed toward the regulatory roles of non-coding RNAs in PD pathogenesis—particularly the interplay between circular RNAs (circRNAs) and microRNAs (miRNAs). Emerging evidence indicates that circRNAs can act as competing endogenous RNAs (ceRNAs), modulating gene expression by sequestering miRNAs and thereby mitigating miRNA-mediated repression of target mRNAs. In this study, we performed a dynamic transcriptomic analysis across four PD stages using RNA-seq data to identify differentially expressed circRNA–miRNA–mRNA networks. We constructed stage-specific ceRNA networks by selecting positively co-regulated circRNAs and linear transcripts that were co-expressed exclusively within the same disease stage. Among the upregulated circRNAs with predicted ceRNA activity, *circPRDM2* and *circHSH2D* were identified as uniquely expressed in PD patients. Additionally, we assessed the coding potential of the predicted target genes to further elucidate the regulatory impact of circRNAs on mRNA expression. Our findings provide new insights into the post-transcriptional regulatory mechanisms involved in PD and highlight candidate stage-specific ceRNA axes that may serve as potential biomarkers or therapeutic targets.

**Supplementary Information:**

The online version contains supplementary material available at 10.1186/s13041-025-01262-2.

## Introduction

Parkinson’s disease (PD) is a neurological disorder that has been found to be the second most common neurodegenerative disease in epidemiological studies [1]. Prevalence of PD is predicted to increase by 76% from 2021 to 2050 based on a modelling study published in 2025 [2]. The clinical diagnosis predominantly relies on the assessments of motor movements, including bradykinesia, and non-motor movements such as cognitive disorder [3]. Advanced imaging and sequencing techniques effectively indicate that the progressive loss of dopaminergic neurons in specific brain regions of substantia nigra, accumulation and phosphorylation of ɑ-synuclein (ɑ-syn) in Lewy bodies are common biomarkers in PD [4]. Additionally, dopamine (DA) metabolites such as reactive oxygen species and DA quinone induce oxidative stress, protein misfolding and functional dysregulation which are closely associated with the impairment of dopaminergic neurons [5, 6]. It has been identified that the toxic metabolites could trigger neuronal loss and lead to the mitochondrial and lysosomal dysfunction in PD. Recent studies highlighted the stress of neuronal activity brought by the irregular forms of ɑ-syn (encoded by *SNCA* gene), including insoluble oligomers and polymorphic amyloid fibrils [7, 8]. Kinetic and biophysical data revealed the surface-catalysed secondary nucleation, a process that catalyses multiple proliferation of new fibrils via the binding of monomers to the fibrils during the aggregation [8]. A previous study revealed a correlation between ɑ-syn and dopamine expression when a decrease of dopamine transporter occurred in the ɑ-syn deficit mouse model [9]. Based on these two established biomarkers in PD, the immune system and inflammatory responses play crucial roles in neuronal dysfunction. Among the activation loop whereby ɑ-syn aggregation stimulates glial cells, proinflammatory cytokines and microglia, interferon-gamma (IFN-γ) plays a pivotal role in promoting neurodegeneration during PD pathogenesis [10, 11].

Studies concerning PD’s pathogenesis and progression have been conducted through the combination of experimental and computational analysis over decades. By utilising algorithmic modelling, machine learning, and statistical inference, computational approaches enable researchers to identify and characterise genetic variations and regulatory interactions. In genetic studies of complex neurodegenerative disease, computational methods are indispensable for multi-omics integrations, facilitating the association of genetic alterations with transcriptomic, proteomic, and epigenomic outcomes [12–14]. These in silico findings generate hypotheses that can be validated experimentally and uncover the underlying molecular mechanisms of diseases.

MiRNAs (18–22 bp) are post-transcriptional regulators that target RNA transcripts by partially complementarily binding to the microRNA element responses (MREs) located within the RNA transcripts. Circular RNAs (circRNAs) and messenger RNAs (mRNAs) can be classified as competing endogenous RNAs (ceRNAs) according to their nature of harbouring the MREs of multiple miRNAs [15]. It has been proven that circRNAs molecules can be specific to certain tissues, disease and developmental stages, and are particularly highly abundant in the brain [16–18]. The circular conformation of circRNAs, formed via back-splicing results in circRNAs being evolutionarily conserved due to their nature of being resistant to 3’-5’ exonuclease RNase R [17]. Sang et al. [19] found that *circSNCA* sponged miR-7 to increase the expression of its homogenous mRNA SNCA along with an increase of cell apoptosis. A recent RNA-seq study demonstrated that *circSV2b* played a role as a miRNA inhibitor interacting with miR-5107-5p to restore the expression of *Foxk1* [20]. As *Foxk1* positively activated Akt1 transcription, which has been identified to regulate cellular responses such oxidative stress, overexpression of *circSV2b* would mediate an interlinked ceRNA-Akt1 axis for resistance of oxidative stress and protection against the loss of DA neurons in PD mice model. Researchers from the Parkinson’s Progression Marker’s Initiative (PPMI) study group have annotated and predicted circRNAs, miRNA and mRNAs using the data from the PPMI study cohort, reinforcing the significance of these RNA molecules in PD patients [12, 21].

In this study, we aimed to use whole blood RNA-seq data from PPMI study cohort to computationally predict the differential expression (DE) of circRNAs and linear transcripts as well as the circRNA-miRNA-mRNA axis based on the DE circRNAs and linear transcripts.

## Materials and methods

### Data retrieval and RNA-seq data quality filtration

RNA-seq data were retrieved from the PPMI (https://www.ppmi-info.org/) for both PD and control cohorts (accessed in June 2024) [22, 23]. The initial PD and healthy control datasets were obtained from Phase 1 stage along with a total of 1,601 subjects prior to quality control filtering, with four replicates included for each individual subject. Whole-blood RNA-seq was designed for PD subjects that had been diagnosed within two years before the longitudinal monitoring of disease progression, and the selected PD patients had to be untreated with any PD medications, measured with Hoehn and Yahr staging evaluation < 3 etc. To further confirm the eligibility of the PD patients for being involved in this study, dopamine transporter (DAT) imaging or vesicular monoamine transporter (VMAT-2) imaging was conducted on all PD candidates. Cognitive and behavioural assessments were conducted at 12-month intervals on all participants and diagnosed subjects, while the blood samples were collected at 3-month intervals during the first 12 months followed by collections in 6-month intervals. Montreal Cognitive Assessment total score was measured towards all healthy control subjects. A detailed description of the RNA-seq data generation from whole blood from the participants can be found in the PPMI publication [23].

Raw RNA-seq data in FASTQ file format from PPMI were trimmed using Trimmomatic v0.39, and their quality was assessed using FastQC v0.11.9 [24, 25]. The STAR v2.7.6a software was used to map and annotate the raw data to hg38 using the reference genome GENCODE v43 [26, 27].

Phase 1 datasets were filtered based on the criteria that GC content from FastQC output must be within the 45–60% range, and the unique mapped reads alignment rate from STAR should be at least 85%. Resultantly, the final datasets consisted of 325 PD samples (Baseline (BL) stage [0 month]: 83 samples; v04 stage [12 months]: 99 samples; v06 stage [24 month]: 83 samples; v08 stage [36 months]): 60 sample and 112 control samples regardless of subjects’ replicates. Of note, number of months indicated the duration that the patients were enrolled into the PPMI program, and their disease progression were re-assessed and recorded.

### Identification of circRNAs and linear transcripts

CircRNAs were identified and annotated by CIRCexplorer2 v2.3.8 through parsing the starChimeric.out.junction output file from the STAR alignment. BWA v0.7.17 was used to align raw data to hg38, which was followed by running the CIRI2 v2.0.6 software for circRNA identification [28–31]. Back-space junction (BSJ) read counts (CIRI2: *junction_reads,* CIRCexplorer2: *readnumber*) was referred to count the number of circRNAs. Each BSJ coordinate (chr:star-end) was treated as a single circRNA. If a gene harboured multiple BSJs, there were analysed individually. Common circRNAs identified by CIRCexplorer2 and CIRI2 were merged for further analysis.

Alignment and assembly of linear transcripts were performed using HISAT2 v2.2.0 and StringTie v1.3.4d, respectively. The preDE.py provided by StringTie generated a table that contained counts per million (CPM) by utilising the coverage values detected from the output of *stringtie -eB* command [32, 33].

### Prediction of differentially expressed circRNAs and mRNAs

In an R v4.3.1 environment, the edgeR v4.0.16 package was used to normalise the library sizes using Trimmed Mean of M-values (TMM) normalisation method. The TMM method trimmed away extreme log fold changes of the data to adjust the differences in sequencing between samples and performed differential expression (DE) testing from the estimated biological variance [34, 35]. Missing calls were replaced with zeros by TMM normalisation method. R-package limma v3.58.1 facilitated voom transformation to stabilise the mean–variance relationship in RNA-seq count data and then fits a linear model to gene expression across samples, as well as the integration of Empirical Bayesian method, enabling stable results from the microarray data [36–38]. Volcano plots were generated by ggplot2 v3.5.1. Differentially expressed (DE) mRNAs were identified using the same methods. DE circRNAs and DE linear transcripts were generated for each stage of PD (|log (fold change)|> 0.3 and p.value < 0.5).

### Gene ontology enrichment and kyoto encyclopedia of genes and genomes analysis

Functional profiles including Gene Ontology (GO) enrichment and Kyoto Encyclopedia of Genes and Genomes (KEGG) pathway enrichment analysis of all DE circRNAs and mRNAs were analysed and plotted by clusterProfiler v4.10.1 and ggplot2 v3.5.1 based on their relevant gene names that have been annotated in org.Hs.eg.db [39].

### Prediction of circRNA-miRNA-mRNA interaction

CircRNA-miRNA interaction was predicted by Circr, which integrated established miRNA-target interaction algorithms including miRanda v3.3a, RNAhybrid v2.1.2, and TargetScan7 under default parameters, thereby leveraging complementary predictive strengths [40–43]. Predictions from all three algorithms were annotated by seed match type and merged into a unified table. Reference miRNA sequences used by Circr was from miRbase. CircRNAs that were predicted to have at least seven miRNA response elements (MREs) were filtered. CircRNA-miRNA axes were selected to scan miRNA-mRNA interaction via the miRWalk database, which integrated data from miRDB and miRTarBase databases [43–46]. Correlations between circRNAs and mRNAs that expressed from PD samples were analysed using Spearman correlation analysis [47]. CircRNA-mRNA correlations at different stages were visualised using heatmaps in Rstudio. The coding ability was retained from Ensembl Canonical (https://www.ensembl.org/index.html). After merging the circRNA-miRNA and miRNA-mRNA axes, the circRNA-miRNA-mRNA network was constructed and visualised using Cytoscape v3.10.0 [48].

## Results

### Data filtration

**Fig. 1:**
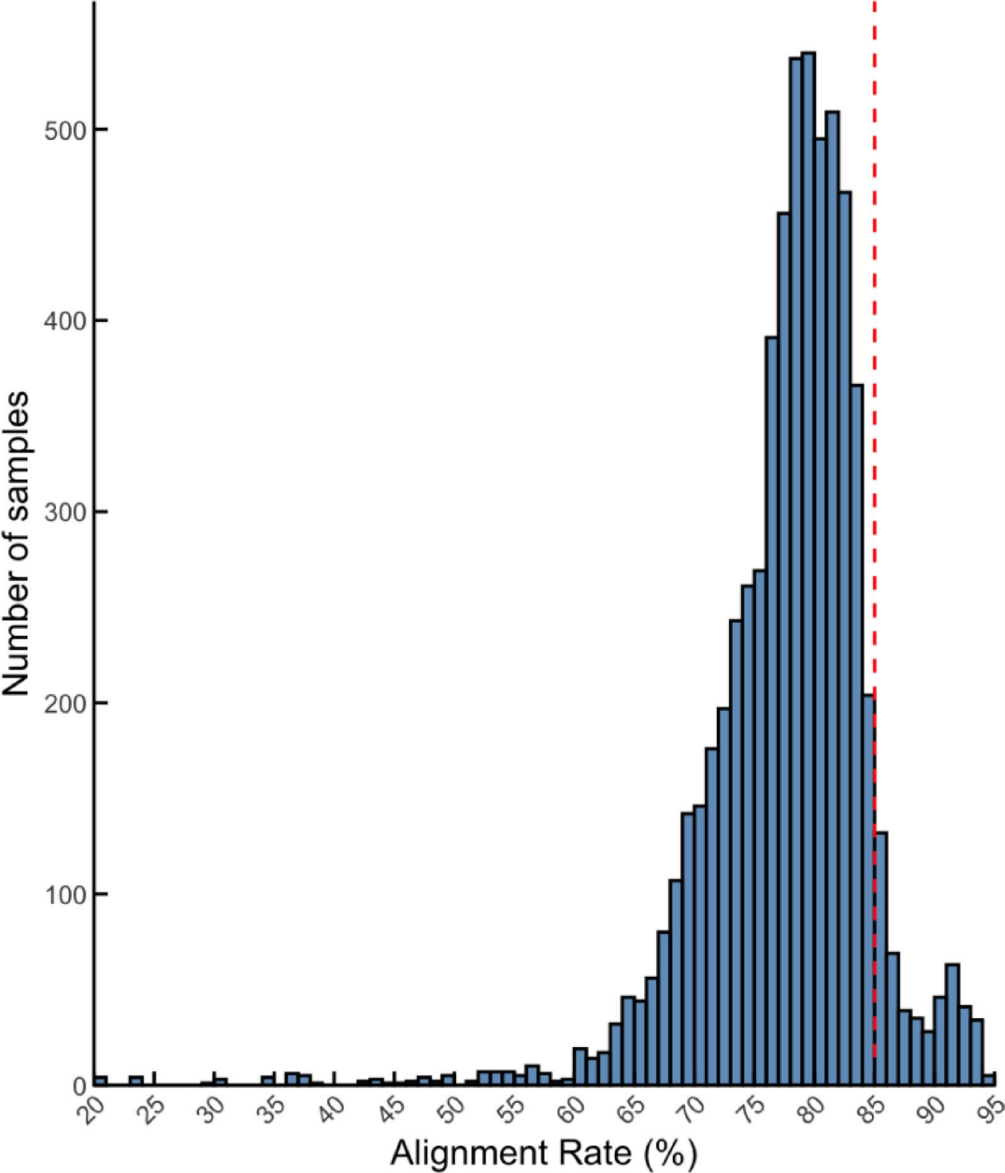
Distribution of alignment rates (%). The red line indicates the cut-off of the STAR alignment rate for high-quality filtration

A total of 12,808 paired-end RNA-seq reads were initially collected and downloaded from PPMI. Quality control was performed using FastQC to assess per-sample read quality, and 12,564 reads were detected with the GC content in between 45 and 60%. Among the filtered samples, the STAR alignment cut-off rate was set up to at least 85% (Fig. 1). The final 325 PD paired-end samples were identified to match the filtering criteria (BL stage: 83 samples; v04 stage: 99 samples; v06 stage: 83 samples; v08 stage: 60 sample) (Table S1 and S2).

### CircRNAs identification

**Fig. 2:**
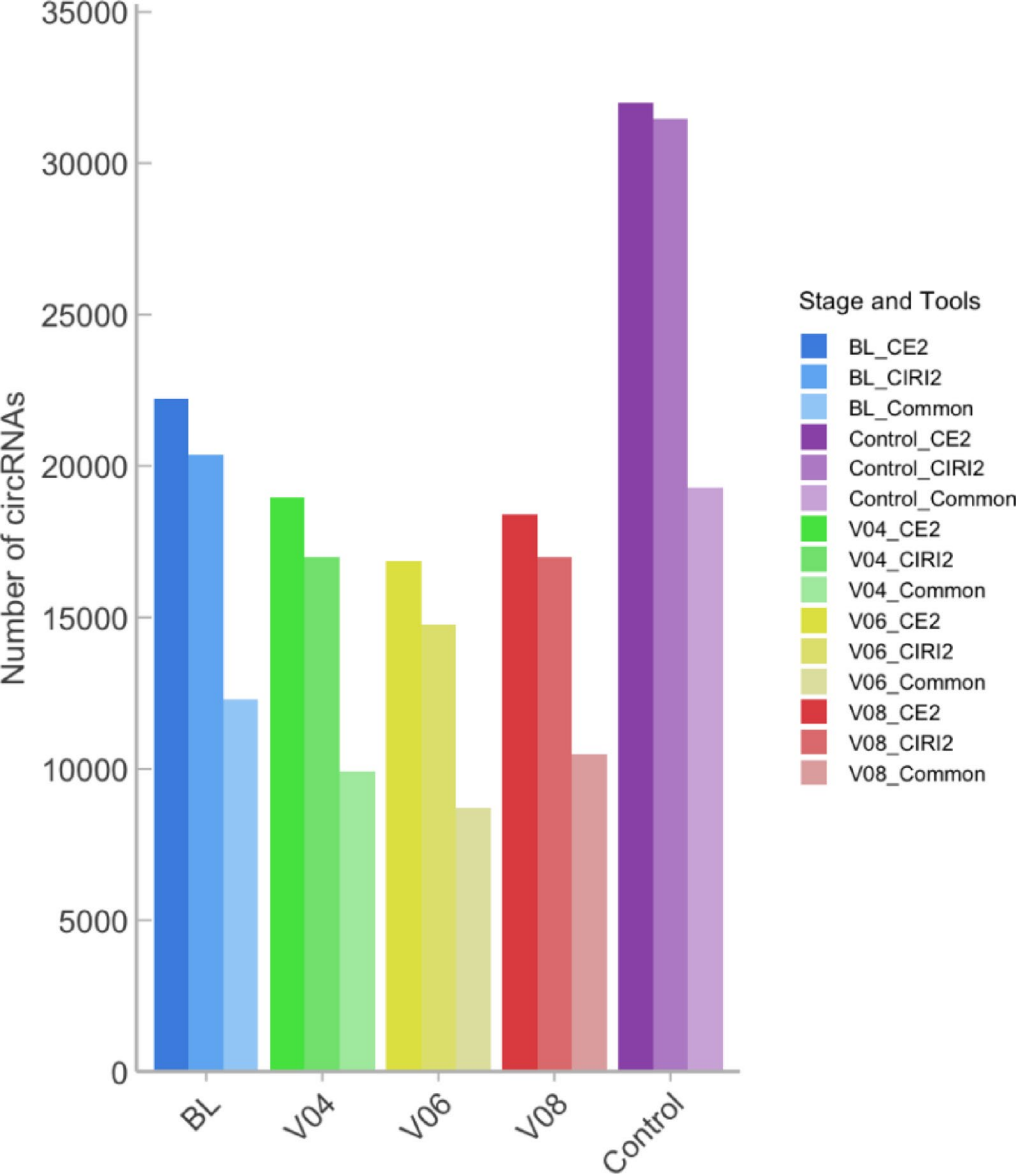
Quantification of predicted and annotated circRNAs. Four disease stages categorised by PPMI were presented, from BL (0 month), V04 (12 months), V06 (24 months) to V08 (36 months), and healthy control samples. CircRNAs were detected by CIRCexplorer2 (CE2) and CIRI2, and the common circRNAs were merged for each condition

CircRNAs identified by both circRNA identification tools with overlapping genomic coordinates were retained only once per individual condition. Non-redundant lists of circRNAs were generated for graphical presentation in Fig. 2. A total of 12,317 common circRNAs were identified in BL stages, with 9,923 at V04 stage, 8,706 at V06 stage, and 10,465 at V08. There were 19,277 common circRNAs identified in the control health samples (Table S3–S17).

### Differentially expressed circRNAs and linear transcripts

**Fig. 3:**
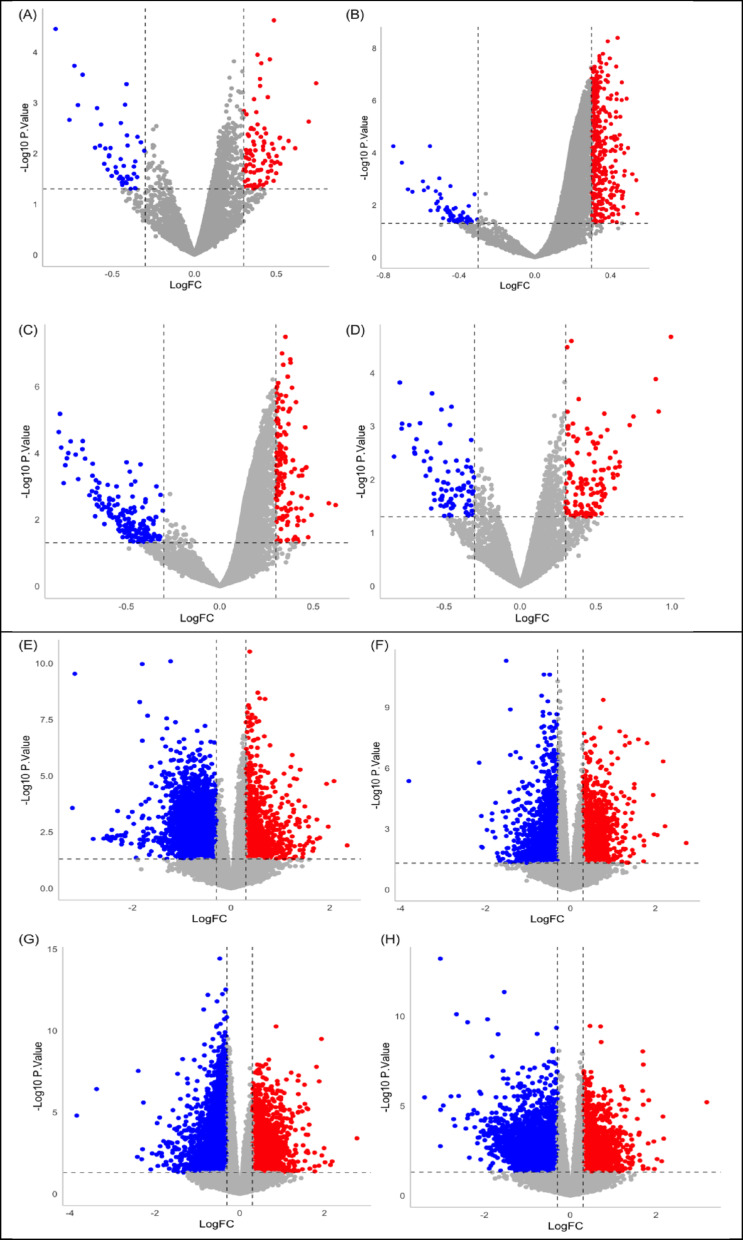
Volcano plots of DE circRNAs and linear transcripts. Down-regulated circRNAs (**A**–**D**) and linear transcripts (**E**–**H**) were presented in blue, while the up-regulated expression was shown in red (*p*.value < 0.5; |logFC|> 0.3)

In order to explore the DE pattern of circRNAs and mRNAs at different disease stages, we applied counts per million (cpm) into edgeR and limma packages for potential expression prediction (|log (fold change)|> 0.3 and p.value < 0.5) (Fig. 3). V04 stage exhibited the most up-regulated circRNAs with a total number of 408 circRNAs, while 173 down-regulated circRNAs were shown at V06 stage as the result of the most down-regulated circRNAs. BL stage exhibited the most down-regulated mRNAs with a total number of 13,186, while the V06 stage presented the highest number of up-regulated mRNAs (4,459). More DE linear transcripts were shown comparing to the DE circRNAs across all four monitoring stages. Table S18–S21 display the DE circRNAs and Table S26–S29 display the DE linear transcripts.

### GO and KEGG enrichment analysis

**Fig. 4:**
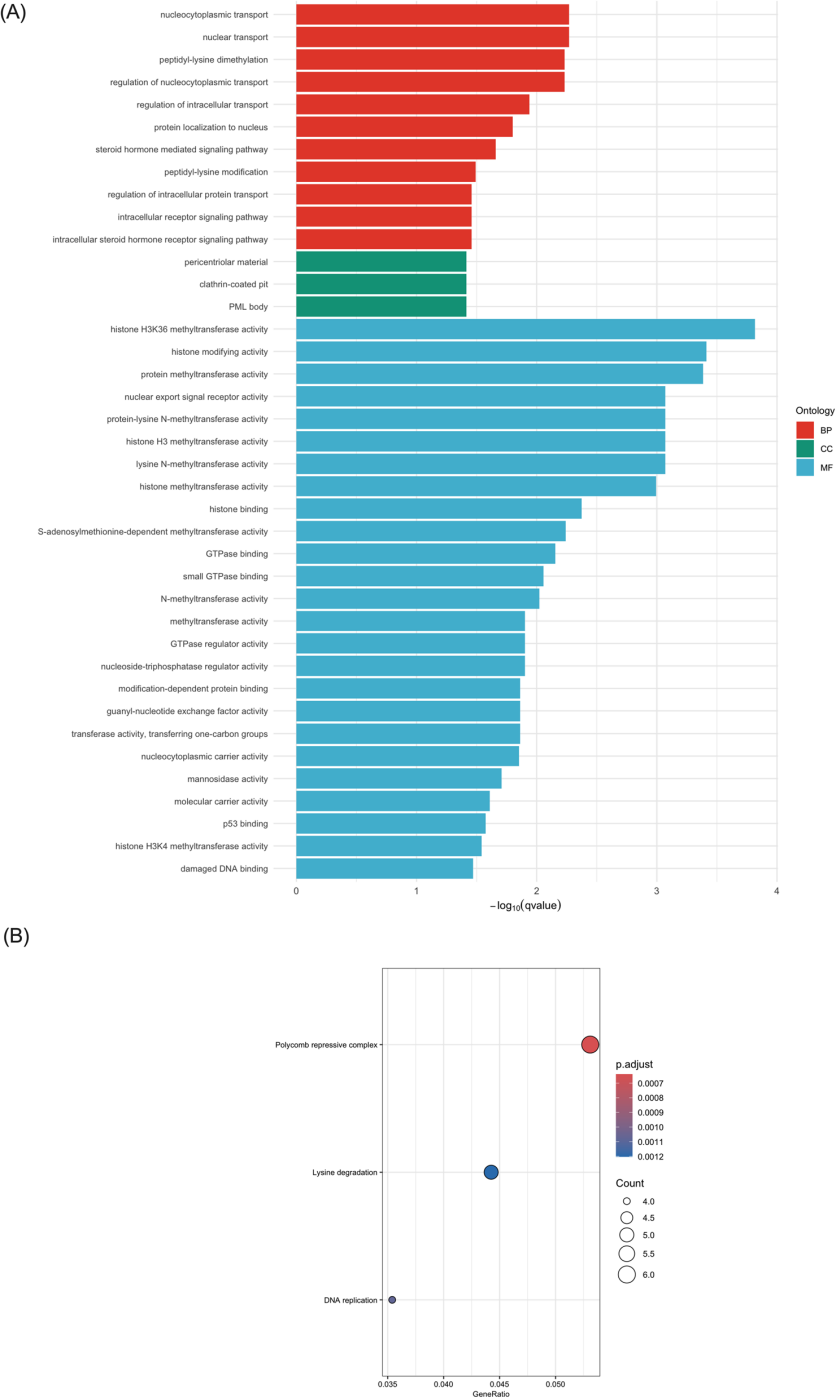

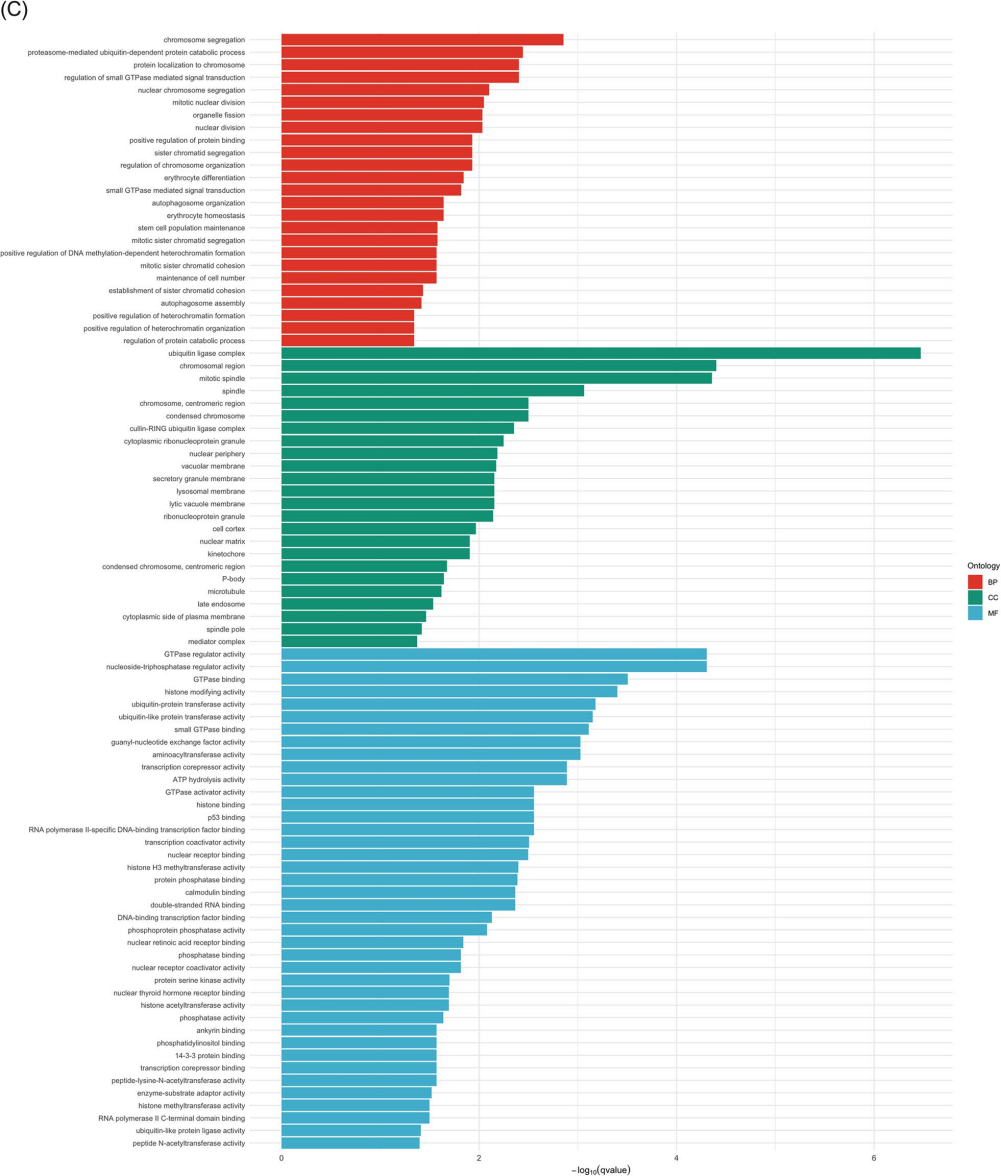

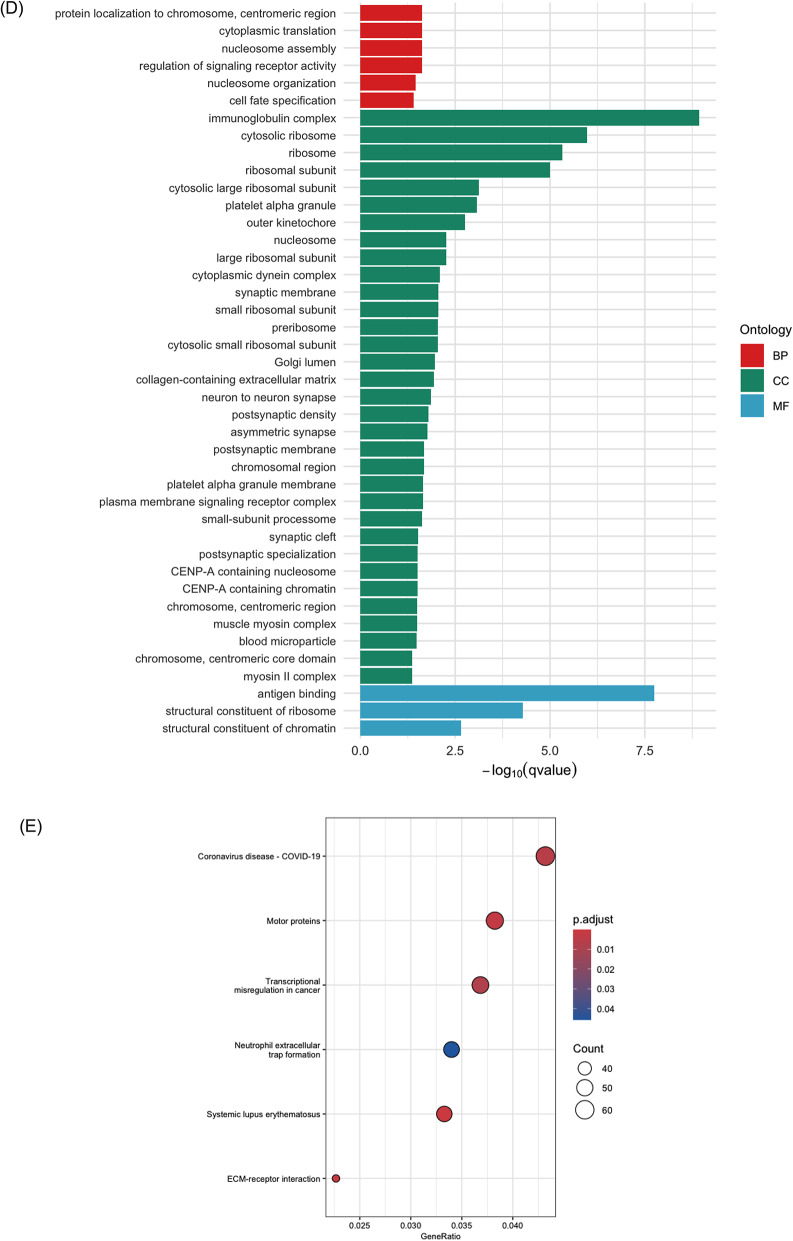

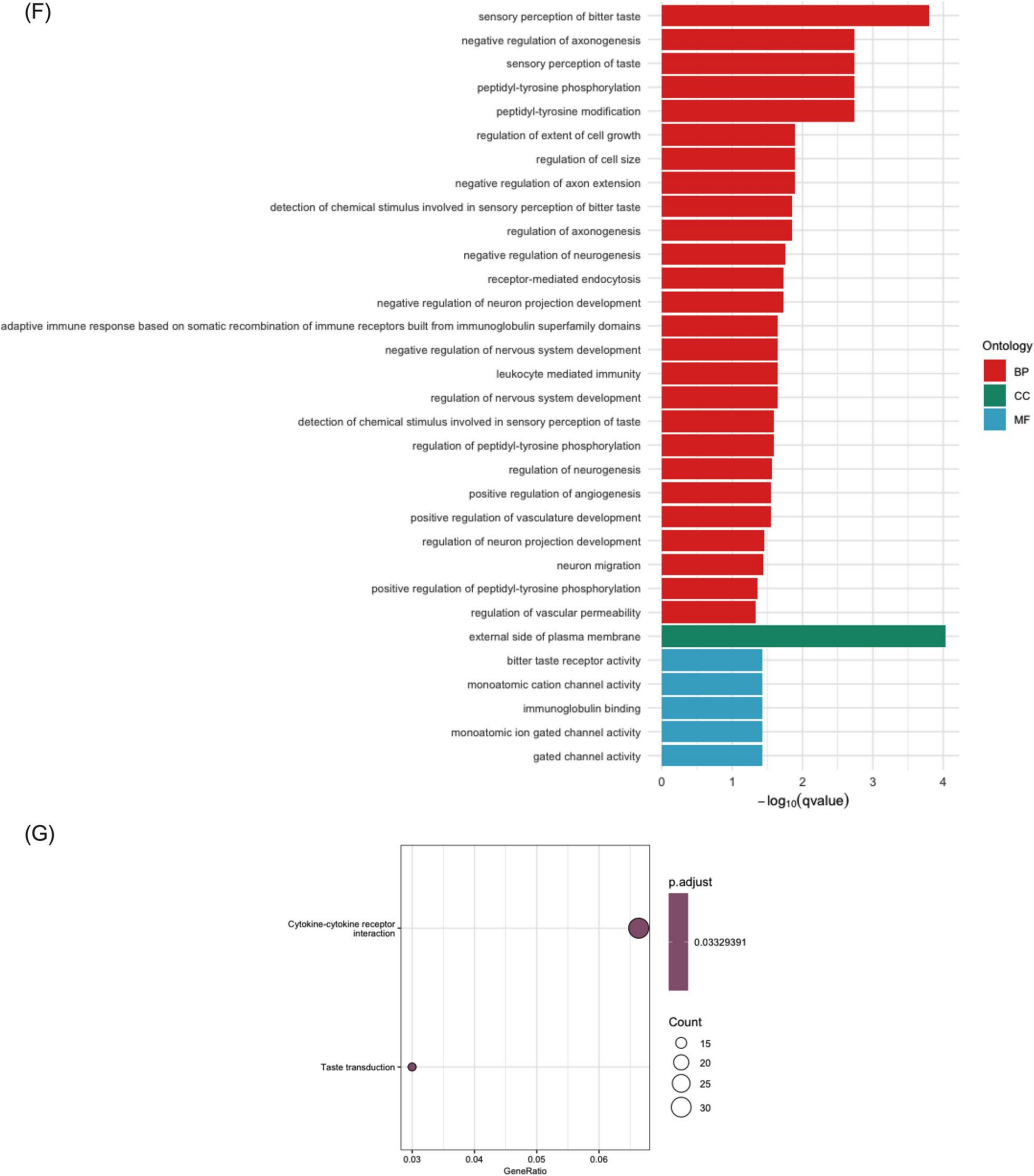
GO and KEGG enrichment analysis of DE circRNAs and linear transcripts. **A**–**C** represented by the genes for down- and up-regulated circRNAs, respectively. **D**–**G** represented the genes for down- and up-regulated linear transcripts. Bar plots and dot plots represented the GO terms and KEGG analysis

GO and KEGG enrichment analysis were performed to explore the pathways and functions that DE circRNAs and target genes may contribute to PD using all parent genes of the DE circRNAs and linear transcripts presented from the volcano plots (Fig. 4). Parent genes encoded for DE circRNAs were enriched for GO terms that commonly associated with epigenetic regulation and transcription, intracellular transport, and cytoskeletal dynamics, as well as cell cycle and genome maintenance (Fig. 4A and C) (Table S22 and S23). Of note, GO terms were constructed based on information concluded by their parent gene and linear transcripts, which only provided provision of potential functions of circRNAs. Figure 4B demonstrates that down-regulated circRNAs participated in polycomb repressive complex in gene regulation. This KEGG enrichment was closely relevant to the enriched GO terms. KEGG pathway analysis showed lysine degradation and DNA replication processes. Up-regulated circRNAs enriched cell cycle for KEGG analysis only (GeneRatio: 13/240; p.adjust = 0.0003). Enriched GO terms by linear transcripts clustered to ribosomal and translation machinery, chromatin, synapse and neuronal structure, immune response and inflammation, sensory and membrane signalling, neurodevelopment and axon guidance, and phosphorylation (Fig. 4D and F) (Table S30 and S31). KEGG analysis revealed that both down- and up-regulated linear transcripts were enriched in pathways associated with the inflammatory and immune responses, as well as in disease-related pathways that may share common signalling mechanisms. Several enriched pathways involved motor proteins implicated in axonal transport and neuronal integrity as neurophysiological function in PD pathogenesis (Fig. 4E and G).

### Prediction of circRNA-miRNA-mRNA network

**Fig. 5:**
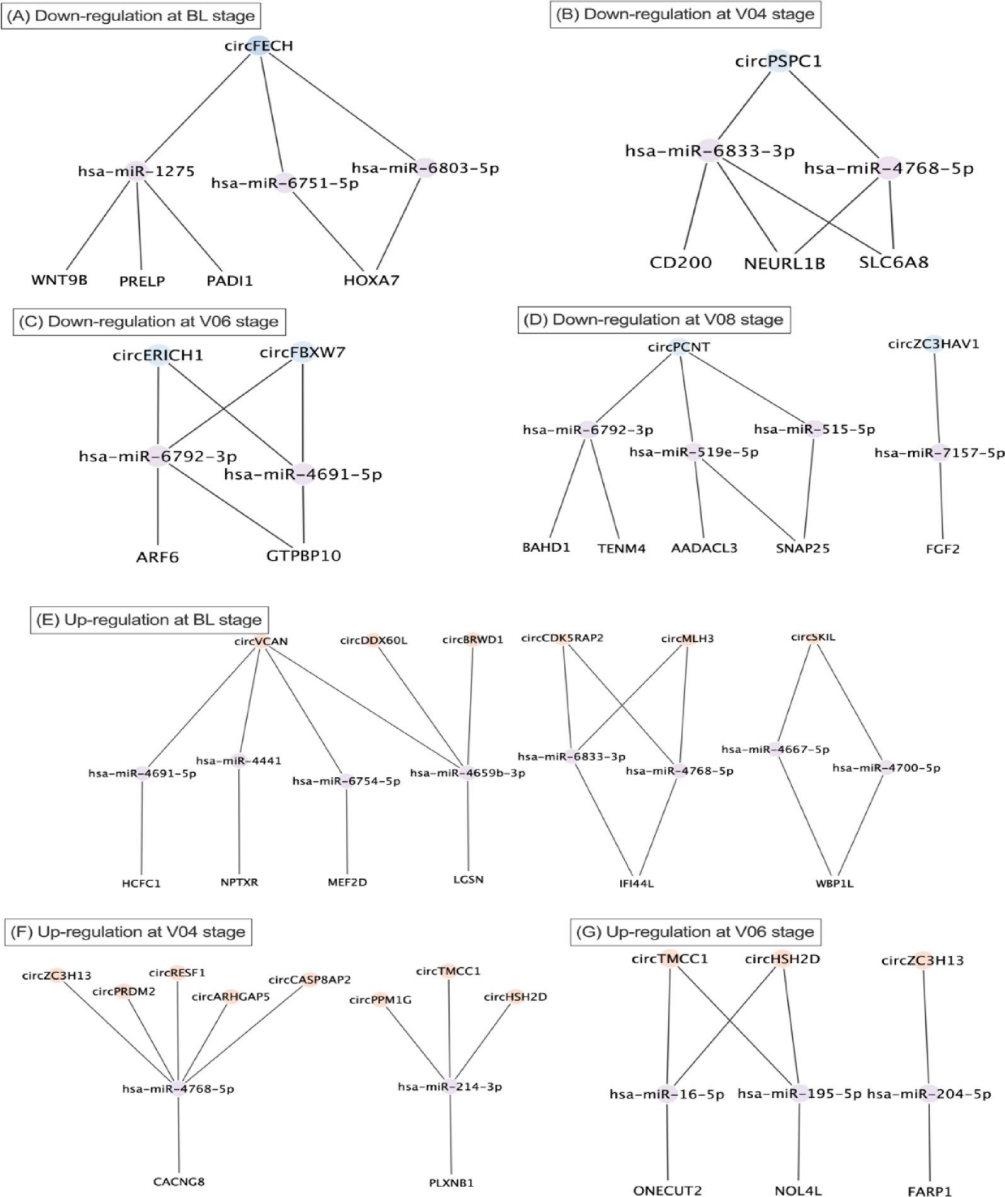
Network of predicted differentially expressed (DE) circRNA-miRNA-DE mRNA. All circRNAs and mRNAs shown in this figure are differentially expressed. **A**–**D** show down-regulated circRNA-miRNA-mRNA network, while **E**–**G** show up-regulated circRNA-miRNA-mRNA network for the four PD stages. V08 exhibited no significantly up-regulated ceRNA-miRNA interactions

The DE circRNAs and mRNAs were selected to predict the circRNA-miRNA-mRNA network. Circr was used to predict the circRNA-miRNA interaction, and the output miRNAs with at least seven binding sites were chosen to scan the miRNA-mRNA interaction via the miRWalk database. From the miRWalk output, only the predicted target mRNAs, retrieved from the DE linear transcripts (Fig. 3E–H), that exhibited positive correlation with the expression of DE circRNAs were retained for circRNA-miRNA-mRNA network prediction (Fig. 5).

**Fig. 6:**
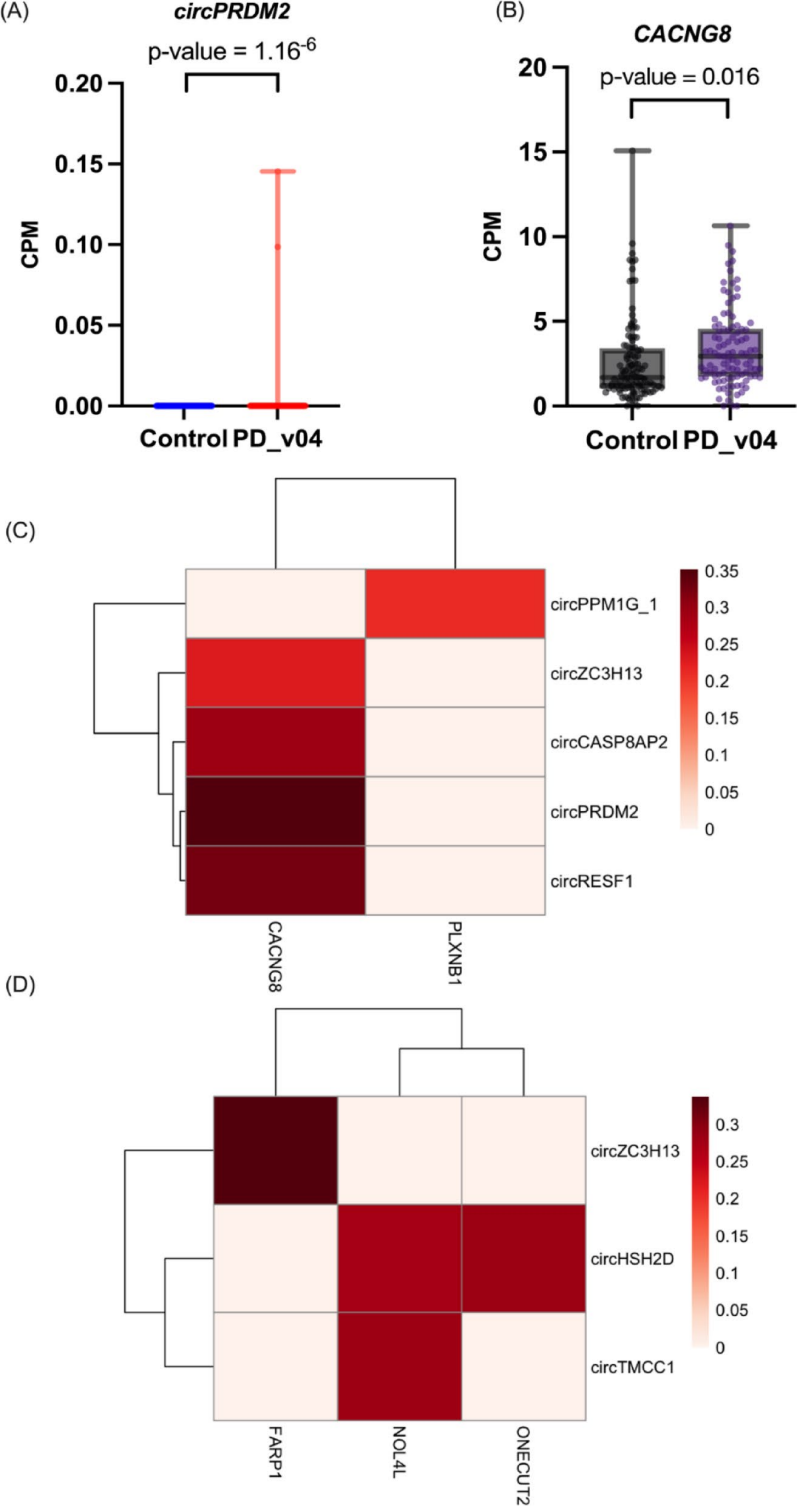
Correlation analysis including uniquely expressed circRNA and its targeting mRNA and statistically significant correlation. **A**–**B** circPRDM2 was uniquely up-regulated while its potential downstream target CACNG8 was also up-regulated at V04 stage. From the box plots, x-axis represents the sample stages, and the y-axis represents their expression in count per million (CPM). **C**–**D** Spearman correlation analysis revealed nine statistically significantly positive correlations of the predictive networks between up-regulated circRNAs and mRNAs at stages V04 and V06 (rho > 0, p-value < 0.05)

To further investigate the potential interactive networks between circRNAs and mRNAs, we performed correlation analysis across PD samples. At stage V04, we observed that *circPRDM2* was uniquely up-regulated in parallel with its predicted downstream target mRNA. Spearman correlation testing also revealed their positive correlation (rho > 0, p-value < 0.05) (Fig. 6). Correlation analysis revealed nine positive correlations with statistical significance at stages V04 and V06.

## Discussion

As PD incidences continues to rise worldwide, there is an increasing number of public PD cohort studies to investigate the pathogenesis and underlying molecular mechanisms [23, 49]. The commonly used RNA-seq techniques enable the rapid research of the transcriptome and RNA molecules by comparing the expression difference between the PD patients and healthy controls. Increasing evidences support that the regulatory functions of circRNAs and miRNAs allow these RNA species to interact and participate in the regulation of target mRNAs and their corresponding protein levels in PD [50]. Therefore, this study revealed the DE circRNAs and linear transcripts in PD, and the potential outcomes delivered by its related predictive circRNA-miRNA-mRNA axis in PPMI cohort.

Firstly, we performed filtration on the RNA-seq datasets by applying a highly stringent criteria (GC content: 45% ≤ samples ≤ 60%; STAR alignment rate: 85% ≧ samples) to obtain the highest quality and most appropriate sequences for all downstream analyses (Fig. 1). As circRNAs calling rely on a small subset of back-splice junction (BSJ) reads, high multi-mapping can inflate false positives and distort normalisation. A low rate can indicate issues suchas adapter contamination, a high proportion of short or ribosomal RNA fragments, or a small or repetitive reference genome. We therefore selected STAR uniquely mapped rate with greater and equal to 85% to reflect the high-quality of RNA template preparation and sequencing, reduce multi-mapping noirse feeding CIRI2 and CIRCexplorer2, and stabilise TMM normalisation in edgeR for sparse BSJ counts [51]. We annotated and predicted circRNAs and linear transcripts according to the monitoring phases, and differential expression was performed for each condition (Fig. 2). More statistically significantly DE circRNAs and linear transcripts (p.value < 0.5; |logFC|> 0) were identified after the baseline (BL) stage which was the beginning of the monitoring, indicating a trend of incremental dysregulation of RNA molecules in PD pathogenesis (Fig. 3). This trend has also been confirmed in a recently published study, as they performed their analysis based on different analytical pipelines using the same PPMI dataset [52]. Of note, Shrestha and Leier [52] applied the filtration criteria with a ≥ 70% genome alignment rate, whereas we selected the PPMI datasets with higher stringency (Fig. 1).

Since GO terms and KEGG enrichment demonstrate the enriched terms based on the functions of the known genes, mRNAs and proteins, the enriched terms for potential function of circRNAs would be derived from their parent gene names. Regarding to the functions of circRNAs, it is well established that circRNAs can specifically regulate the expression of their parental genes and acting as miRNAs sponges to regulate the downstream mRNAs expression [16, 53]. In our study, while the relationship of a proportion of identified circRNAs and their parent genes remains unelucidated, it would be biologically significant to present the functions of their parent genes. Moreover, the description on the functions of the target mRNAs that were predicted to be regulated by circRNAs has also been developed based on the multiple facets of circRNAs. GO enrichment analysis was consequently applied to address the biological functions of the circRNAs and mRNAs that were involved in the network (Fig. 5). Among the down-regulated circRNAs identified within the ceRNA network, those derived from parent genes *FBXW7* and *PCNT* were enriched in the regulation of intracellular transport. This finding emphasised that disruption of intracellular transport between organelles within cells can lead to accumulation of misfolded proteins, such as the significant biomarker ɑ-syn [54, 55].

There were more GO terms enriched by the parent genes of the up-regulated circRNAs. From our enrichment analysis, ATP hydrolysis activity was enriched by *DDX60L and MLH3*, several studies have demonstrated the significance of mitochondrial ATP production and PD biomarker. For instance, the burst dopaminergic neurons serve as electron resources into complex IV during the electron chain transport to drive mitochondrial membrane potential and ATP production, while ɑ-syn would decrease complex I activity and reduce ATP production [56, 57]. Parent genes *CDKRAP2*, *MLH3* and *TMCC1* enriched a group of GO terms that can be clustered into cell cycle and chromosomal mitosis, which including chromosome segregation, mitotic nuclear division, chromatid segregation, and nuclear division. Although these genes and their corresponding circRNAs have not been extensively investigated in PD, the potential association between cell cycle and circRNAs in PD has been highlighted in this predictive analysis. A number of previous studies have revealed the abnormal regulation of cell cycle and PD progression. Höglinger et al. [58] revealed the mitosis-associated proteins E2F-1 transcription factor were activated and its chromosome aberrantly duplicated in mature dopaminergic neurons from PD patients. Post-mitotic neurons, also known as senescent cells that have permanently exited the cell cycle, are particularly vulnerable to degeneration in PD [59]. In aged PD patients, the loss of chromatin-organising factor could mediate the senescence program in post-mitotic dopaminergic neurons, consequently secreting local inflammatory responses [60]. Epigenetic modification indicates the change of gene expression or function without direct alteration in DNA sequences. This process can be characterised by DNA methylation, post-transcriptional modifications of histone, and gene dysregulation mediated by the non-coding RNAs. We also identified that up-regulated circRNAs were enriched for terms associated with histone methylation at arginine or lysine N-terminal residue, which are key mechanisms in epigenetic modulation of gene expression [61]. In a previous PD study, H3K4me3 and H3K27me3 which are promoting and repressive epigenetic modification histone signals, were observed to regulate *SNCA* through post-translational modification, thereby controlling the accumulation of ɑ-syn protein encoded by *SNCA* [62]. The comprehensive enrichment of epigenetic regulatory terms highlighted a multifaceted network of chromatin-associated mechanisms that were likely disrupted in PD.

In addition to the cellular component aspects for up-regulated circRNAs, the identification of microtubule-related terms reflected the destabilisation and fragmentation of microtubule cytoskeleton (Fig. 4, Table S23). Since microtubules are essential for maintaining cellular morphology and facilitating axonal transport, their impairment can significantly contribute to cellular damage and neuronal dysfunction [63]. In our study, *ARHGAP5* derived circRNAs enriched GTPase activities to reveal regulatory roles in the protein switching mechanism in PD. GTPases are enzymes that hydrolyse active guanosine triphosphate (GTP) bound state to inactive guanosine diphosphate (GDP) bound state, thereby regulating vesicle trafficking and cytoskeletal organisation [64]. Based on this concept, GTPases, particularly Ras-associated binding (Rab) GTPases, have been widely discussed. For example, mutations in the LRRK2 phosphorylation sites for Rab35 increases ɑ-syn aggregation and fibrils for neurotoxicity [65]. This evidence collectively suggests the dysregulation of cellular processes, potentially mediated by the up-regulation of circRNAs expression, may contribute to the progression of PD.

Among the GO terms enriched by the linear transcripts (Fig. 4D and F), cell fate specification refers to the undifferentiated cells being directed to specific lineages. Neuronal and glial differentiation of embryonic stem cells-derived neural progenitor is activated by the growth factor *FGF2,* additionally showing the development of neuronal-specific factors and differentiation into neuronal lineage [66]. SNAP25 is a member of soluble N-ethylmaleimide-sensitive factor attachment protein receptor (SNARE) complex. Disruption of the linear transcript SNAP25, which was down-regulated in our predicted network, was found to be associated with abrupt fusion of synaptic vesicles with the pre-synaptic membrane and dysregulation the synaptic transmission in PD [67]. The up-regulated *CACNG8* enriched ion channel activities as it encodes for TARP γ-8 protein for calcium voltage-gated channel, which is responsible for basal expression, trafficking and localisation of AMPA receptor on synaptic dendrites [68]. Although *PLXNB1* remains unexplored in PD pathogenesis, its protein function plays crucial roles in axonogenesis, cellular communication and cytoskeleton dynamics with activation of Ras-GAP domain via small GTPases in Alzheimer’s disease [69, 70]. Co-finding of GTPase- and plexins-related GO terms that were enriched by the parent genes of the DE circRNAs and linear transcripts highlighted the functional relationship of these interactive pathways in PD pathology.

Through the investigation of circRNA sponging miRNA and regulation of target gene expression, we predicted circRNA-miRNA interactions that exhibited at least seven miRNA binding sites, combined with the miRNA-mRNA interaction to form an interactive network. To predict circRNA-miRNA interactions, we employed Circr tool, which integrated established algorithm including miRanda, RNAhybrid, and TargetScan, which relied on sequence complementarity between circRNAs and miRNAs [40]. miRanda considered both base-pairing stability and conservation of the miRNA seed sequence, assigning a weighted score based on base matches and gap penalties [41]. RNAhybrid focuses exclusively on the thermodynamic stability of duplexes, reporting the most energetically favourable binding sites [42]. TargetScan searched for canonical seed matches (8mer, 7mer, and 6mer sites) within circRNAs sequences [43]. Circr then collected the raw predictions from the three integrated algorithms, converted to genomic coordinates, and annotated according to the seed match categories [71]. The miRNAs that were predicted to be involved in circRNA-miRNA interactions were used to predict the miRNA-mRNA in miRWalk [44]. Based upon the predicted target genes, we annotated the coding probability of the linear transcript isoforms of the target genes (Table 1). As these linear transcripts had coding potential, these coding mRNAs and their downstream proteins could be affected by presence of circRNAs.

**Table 1:** Predicted target mRNAs and their annotated transcripts in PPMI datasets

Transcript ID^1^	Name	Stages	Dysregulation	bp	Protein	Biotype
ENST00000375471.5	PADI1	BL	Down	3846	663aa	Protein coding
ENST00000343110.3	PRELP	BL	Down	5769	382aa	Protein coding
ENST00000290015.7	WNT9B	BL	Down	4972	357aa	Protein coding
ENST00000242159.5	HOXA7	BL	Down	2016	230aa	Protein coding
ENST00000253122.10	SLC6A8	V04	Down	3931	635aa	Protein coding
ENST00000369800.6	NEURL1B	V04	Down	6427	555aa	Protein coding
ENST00000473539.5	CD200	V04	Down	1524	294aa	Protein coding
ENST00000222511.11	GTPBP10	V06	Down	7489	387aa	Protein coding
ENST00000298316.7	ARF6	V06	Down	3875	175aa	Protein coding
ENST00000278550.12	TENM4	V08	Down	14,381	2769aa	Protein coding
ENST00000416165.6	BAHD1	V08	Down	4779	780aa	Protein coding
ENST00000359318.8	AADACL3	V08	Down	4055	407aa	Protein coding
ENST00000685131.1	SNAP25	V08	Down	2862	206aa	Protein coding
ENST00000264498.9	FGF2	V08	Down	6801	288aa	Protein coding
ENST00000310441.12	HCFC1	BL	Up	8876	2035aa	Protein coding
ENST00000333039.4	NPTXR	BL	Up	5830	500aa	Protein coding
ENST00000348159.9	MEF2D	BL	Up	5912	521aa	Protein coding
ENST00000370657.9	LGSN	BL	Up	5640	509aa	Protein coding
ENST00000370751.10	IFI44L	BL	Up	5829	452aa	Protein coding
ENST00000448841.7	WBP1L	BL	Up	4129	363aa	Protein coding
ENST00000358536.8	PLXNB1	V04	Up	7308	2135aa	Protein coding
ENST00000270458.4	CACNG8	V04	Up	8850	425aa	Protein coding
ENST00000627049.2	FARP1	V06	Up	5103	1076aa	Protein coding
ENST00000621426.7	NOL4L	V06	Up	7023	680aa	Protein coding
ENST00000491143.3	ONECUT2	V06	Up	16,433	504aa	Protein coding

^1^Peptidyl arginine deiminase 1 (PADI1); Proline and arginine rich end leucine rich repeat protein (PRELP); Wnt family member 9B (WNR9B); Homeobox A7 (HOXA7); Solute carrier 6 member 8 (SLC6A8); Neuralised E3 ubiquitin protein ligase 1B (NEURL1B); Cluster of differentiation 200 (CD200); GTP binding protein 10 (GTPBP10); ADP-ribosylation factor 6 (ARF6); Teneurin transmembrane protein 4 (TENM4); Bromo adjacent homology domain containing 1 (BAHD1); Arylacetamide deacetylase like 3 (AADACL3); Synaptosome associated protein 25 (SNAP25); Fibroblast growth factor 2 (FGF2); Host cell factor C1 (HCFC1); Neuronal pentraxin receptor (NPTXR); Myocyte enhancer factor 2D (MEF2D); Lengsin, lens protein with glutamine synthetase domain (LGSN); Interferon induced protein 44 like (IFI44L); WW domain binding protein 1 like (WBP1L); Plxin D1 (PLXND1); Calcium voltage-gated channel auxillary subunit gamma 8 (CACNG8); FERM, ARH/RhoGEF and Pleckstrin domain protein 1 (FARP1); Nucleolar protein 4 like (NOL4L); One cut homeobox 2 (ONECUT2)

In our analysis, correlation analysis revealed nine circRNA-mRNA pairs that exhibited statistically significant positive correlations at stages V04 and V06. These findings suggested that subsets of circRNAs may be functionally linked to the regulation of mRNA expression in PD progression. Although certain number of circRNA-mRNA interactions reached statistical significance under Spearman criteria, it is critical to note that the broadr circRNA-mRNA interaction network also carries biological significance. The lack of statistical correlation in the remaining pairs may reflect factors such as stage-specific expression dynamics or the presence of complex regulatory interactions that are not fully captured by correlation analysis alone.

From the six down-regulated circRNAs and their potential downstream target linear transcripts that were also down-regulated were utilised for predicting the circRNA-miRNA-mRNA networks across the four monitoring stages (Fig. 5). In our samples and predictive pipelines, *circFECH*, *circPSPC1*, *circEIRCH1*, *circPCNT* and *circZC3HAV1* are firstly reported, and an isoform of *circFBXW7* has been observed before. Although the majority of the identified miRNA-mediated ceRNAs have not been extensively investigated before, the following discussion highlights the importance of uncovering these molecules in PD by examining their potential associations with PD pathology. In a previous blood transcriptome analysis, *FECH* was down-regulated and identified as being involved in the iron metabolism in the pathogenesis of PD [72]. *FECH* is produced by the transcription factor GATA-1, encoding the enzyme ferrochelatase, which is essential for the production of heme. Heme is an essential prosthetic group in hemoproteins involved in oxygen transport within the blood. It has been found that *FECH* interacts with *SNCA* which encodes for ɑ-synuclein in the autosomal dominant PD [73]. The *circFECH* sponging miR-1275 was predicted in the miRNA-mediated ceRNA network in the multiple sclerosis [74]. The predicted target gene *WNT9B* by *circFECH* was found with genetic variations in multiple sclerosis, which is also a neuronal disorder [75]. An early-onset Alzheimer’s disease study predicted potential biomarker of *PRELP* attaining a causal relationship with inflammatory states [76]. In the last five years, a growing body of research has identified *PADI1* and its involvement in multiple cancers including pancreatic ductal adenocarcinoma, colorectal cancer, nasopharyngeal carcinoma and several squamous cell carcinomas [77–81]. These findings indicate the underlying regulatory ability of *PADI1* across various biological functions. In gliomas, a type of brain tumour, the telomere-related gene *HOXA7* exhibits significant roles in tumour development [82]. *PSPC1* maintaining genome integrity during the DNA damage response decreased in Alzheimer’s disease research [83]. In stage V04 (12 months) of our PD samples, the down-regulated *CD200* and *SLC6A8* have been investigated with abundant results to include or exclude the relationship with PD pathology before. Manich, Recasens (2019) [84] revealed the enrichment of *CD200* in neurons and different cell types including glial cells, immune cells, and B-lymphocytes, as well as having highlighted the microglia-neuron communication directed by the existence of *CD200*. Later, Wang, Gong (2020) [85] discovered that *CD200* was enriched in the midbrain and the knockout of *CD200* mediated the neuroinflammatory responses and loss of dopaminergic neurons in the substantia nigra. From the *SLC6A8* (encoding for creating transporter) mice model, decrease of cerebral creatine in dopaminergic neurons developed hyperactivity, however, their behavioural abnormalities including motor controls and balance were unrelated to the motor impairment shown in PD [86]. *NEURL1B* was a key gene contributing to a lncRNA-miRNA-mRNA ceRNA network in primary ovarian insufficiency, indicating its regulatory potential within the ceRNA interactions. A previous study identified that overexpression of *circFBXW7* interacts with miR-492 to inhibit proliferation, migration, and invasion of non-small cell cancer cells [87]. In PPMI stage V06 (24 months) DE circRNA, *ERICH1* was found in two other neurological diseases, which are myasthenia gravis and multiple sclerosis [88, 89]. Significantly, hsa_circ_0001451, an isoform of *circFBXW7*, was also down-regulated in the blood sample and related to oxidative stress responses from a previous bioinformatics analysis of PD samples [13, 90]. Oxidative stress is reflected by the imbalance between the production of reactive oxygen species and the process of neutralisation, leading to cellular damage, particularly in DA [91]. This results further supported the potential involvement of *circFBXW7* and its relevant biological disruption of oxidative stress in PD pathogenesis, as well as emphasising the validity of computational analysis. Yang, Gao (2018) [92] reported that *circFBXW7* was abundantly expressed and closely associated with glioblastoma in human brain. *ARF6* was observed as one of the regulators to recruit PIP5K1γ and elevate the expression of the membrane lipid phosphoinositide PI(4,5)P_2_, consequently seeding aggregation of ɑ-syn, augmenting mitochondrial IP_3_-mediated Ca^2+^ release, and eventually modulating neurotoxicity in PD [93]. Biological significance of *GTPBP10* has been analysed in relation to mitochondrial ribosome within a decade [94]. Although *circFBXW7* and *circPCNT* have not been identified in PD previously, Bierne, Tham (2009) [95] firstly reported that *BAHD1* served as formation of heterochromatin and interact with other heterochromatin factors and DNA-bound transcription factors to supress transcription. Based on this finding, increasing number of studies unveil the regulatory roles of *BAHD1* in cell proliferation, differentiation, inflammation, development, and tumour metastasis [96, 97]. Several cases reported the nucleotide variant of *TENM4* was a risk factor for essential tremor from the early-onset PD patients, which highlight the genetic overlap between PD and tremor [98–101]. *AADACL3* was only found in sarcoidosis prognosis [102]. The down-regulated *SNAP25* and *FGF2* in the stage V08 (36 months) have been widely discussed with regards to PD. The *SNAP25* encoded protein was responsible for calcium-dependent exocytosis and a neurotransmitter exhibiting in the synapse, and its down-regulated expression was observed to correlate with dysfunction of SNARE core complex in a PD model [103]. Also, studies revealed *SNAP25* delivers significant association of the neurogranin and cognitive impairment from PD patients [104–106]. Interestingly, a recent advancement in biotechnology engineering successfully reprogrammed and recombined the protease that was originally specific to *SNAP25,* enabling it to selectively cleave ɑ-syn at the “nonamyloid core” region [107]. We revealed a circRNA-miRNA-mRNA network in relation to *circZC3HAV1* and *FGF2.* The overexpression of *FDF2* could cause non-motor symptoms in PD model by increasing the expression of Rab proteins, which are regulators of membrane components and extracellular vesicle release [108]. Another study demonstrated the extracellular vesicles derived from *FGF2*-primed astrocytes acted to protect against the mitochondrial and synaptic toxicities through promoting NCAM which served as neuroprotector [109].

More positively related circRNAs and mRNAs were observed for up-regulated miRNA-mediated axis prediction. From the up-regulated circRNAs in the stage BL (0 months), *circVCAN* positively regulated MEF2C-JAGGED1 axis via sponging miR-488-3p to promote proliferation of glioma cells [110]. This study also reported that *circVCAN* exhibited a more stable form than its linear transcript counterpart, which reinforces the notion that circRNAs have a greater stability. Although the other up-regulated circRNAs have not been investigated in PD before, their ability to sponge miRNAs and regulate target genes has been widely discovered in different diseases. *circBRWD1* demonstrated its ability in modulating the miR-1277/TRAF6 and miR-513a-5p/TNPO1 axes in osteoarthritis and hepatocellular carcinoma respectively [111, 112]. *circMLH3* was identified to participate in the regulation of miR-590-3p/TAK1 expression in sepsis, which is a systematic inflammatory response [113]. The *circSKIL*/miR-532-5p/Notch1 axis was found in the osteoblastic differentiation. Dysregulated *NPTXR* was regulated by hydrogen peroxide which was induced by oxidative stress and damage from PD [114]. The PD patients with and without a *GBA1* mutation revealed a significant change of the protein expression of *NPTXR* in cerebrospinal fluid (CSF) [115, 116]. The potential biomarker of *NPTXR* was also validated in the van Steenoven, Koel-Simmelink (2020) [117] study, which reported that the expression of CSF *NPTXR* was low in PD and even lower in dementia with Lewy bodies. *MEF2D* is a member of the *MEF2* family of transcription factors, which are known for their roles in neuronal differentiation and survival. The study by Gong, Tang (2003) [118] found that *MEF2D* localised in neuronal mitochondria and was targeted by cyclin-dependent kinase 5 (Cdk5), resulting in the inducement of neurotoxins and neuronal apoptosis [119]. It was previously found that the inhibition of miR-421 could restore the function of *MEF2D*, thereby protecting neurons from neurotoxic cascade [120]. This interaction was closely reflected in the findings of this study, as it was predicted that another miRNA, miR-6754-5p, was sponged by *circVCAN* to up-regulate the expression of *MEF2D*. Moreover, Yao, Li (2012) [121] proposed the use of bis(3)-cognition to activate *MEF2D*, which they achieved to ameliorate motor defects by maintaining redox homeostasis and restoring tyrosine hydroxylase signal in the substantia nigra of PD samples. *WBP1L* was previously reported in a multiple sclerosis study, whose findings indicated its participation in the pathogenesis of neurodegenerative diseases such as multiple scelerosis [122]. *CircPRDM2* and *circHSH2D* were statistically significantly uniquely expressed in stage V04 and V06, marking their first report in PD samples. A previous study revealed that *circPRDM2* acted as a molecular sponge and abolished miR-760 of targeting *EZH2* in osteosarcoma [123]. Similarly, *circARHGAP5* was discovered to elevate *Rock1* by sponging miR-29a-3p during nerve injury [124]. These findings suggest that circRNAs may participate in PD pathology through similar regulatory mechanisms involving miRNA sequestration and modulation of disease-relevant targets. Neuronal synaptic plasticity promoted the secretion of proinflammatory mediators and glutamate metabolism in astrocytes by binding *circTMCC1* to the transcription factor; the nuclear factor kappa light chain enhancer of activated B cells (NK-κB), and the p65-CREB complex [125, 126]. Interestingly, the overexpression of miR-314-3p from being sponged by *circTMCC1* was found to inhibit autophagy-related proteins of dopaminergic neurons, and lead to an accumulation of ɑ-syn in PD [127]. On the other hand, miR-314-3p could also interact with the NF-κB pathway as *circTMCC1* did, highlighting the interaction between *circTMCC1/*miR-314-3p in our analysis [128]. The up-regulated *CACNG8*, which was positively regulated by the uniquely expressed *circPRDM2* in a recent transcriptomic study where they found negative influences of cannabinol to ion channels and synaptic transmission, eventually mediating neurological disorders [129]. The three up-regulated circRNAs, *circTMCC1, circHSH2D,* and *circZC3H13,* in V06 were also present in V04, indicating a prolonged dysregulated expression. *ONCUT2* was also predicted as a potential target gene of LINC00269 by a bioinformatics study of the pyroptosis pathway in PD [130]. Two recent neuroblastoma studies revealed the involvement of *NOL4L* including *circPDE5A/miR-362-5p/NOL4L* axis and circ_0132817/miR-432-5p/NOL4L axis [131, 132].

Parkinson’s disease is a highly complex neurodegenerative disorder, in which molecular changes interact with genetic, environmental, and clinical factors. While this study relies on computational predictions to construct and characterise circRNA-miRNA-mRNA regulatory networks in PD, clinical severity, cognitive impairment, or disease progression are necessated to be further assessed to address the pathogenesis. Future research should prioritise experimental validation to confirm the biological relevance of the identified ceRNA axes. Techniques such as RNA immunoprecipitation (RIP), luciferase reporter assays, and qRT-PCR can be employed with longitudinal clinical metadata to verify direct interactions between circRNAs, miRNA, and their target mRNAs. Additionally, the spatial and temporal expression patterns of these RNAs should be assessed in PD-relevant tissues such as brain using single-cell RNA sequencing or in situ hybridisation to uncover cell-type-specific regulatory mechanisms. Beyond validation, functional studies using knockdown or overexpression models in cellular and animal systems will be essential to determine the causal roles of candidate circRNAs in PD. The application of machine learning models trained on experimentally validated ceRNAs could enhance predictive accuracy.

Overall, this study provides a comprehensive analysis of stage-specific circRNA-miRNA-mRNA networks in PD progression, offering new insights into the dynamic role of circRNAs as ceRNA during disease progression. By integrating expression data and predictive interactions, we identified circRNAs that positively regulated dysregulated mRNAs through sequestering miRNAs. These findings underscore the role of circRNA-involved post-transcriptional regulation in contributing to the molecular complexity and regulatory heterogeneity underlying PD. The predicted ceRNA axes represent promising candidates for further experimental validation and may inform the development of stage-specific biomarkers or therapeutic strategies targeting non-coding RNA networks in neurodegenerative diseases.

## Supplementary Information

Additional file1 (XLSX 39383 kb)

Additional file2 (XLSX 34365 kb)

## Data Availability

The datasets used and/or analysed during the current study are available from the corresponding author on reasonable request.
